# A Low-Light Sensor Image Enhancement Algorithm Based on HSI Color Model

**DOI:** 10.3390/s18103583

**Published:** 2018-10-22

**Authors:** Shiping Ma, Hongqiang Ma, Yuelei Xu, Shuai Li, Chao Lv, Mingming Zhu

**Affiliations:** 1 Aeronautics Engineering College, Air Force Engineering University, Xi’an 710038, China; mashiping@126.com (S.M.); yuelei_xu@163.com (Y.X.); lishuailishuai@163.com (S.L.); lvchao1112@163.com (C.L.); ming_paper@163.com (M.Z.); 2 Unmanned System Research Institute, Northwestern Polytechnical University, Xi’an 710072, China

**Keywords:** image enhancement, Retinex model, color model, feature learning, convolutional neural network, batch normalization

## Abstract

Images captured by sensors in unpleasant environment like low illumination condition are usually degraded, which means low visibility, low brightness, and low contrast. In order to improve this kind of images, in this paper, a low-light sensor image enhancement algorithm based on HSI color model is proposed. At first, we propose a dataset generation method based on the Retinex model to overcome the shortage of sample data. Then, the original low-light image is transformed from RGB to HSI color space. The segmentation exponential method is used to process the saturation (*S*) and the specially designed Deep Convolutional Neural Network is applied to enhance the intensity component (*I*). At the end, we back into the original RGB space to get the final improved image. Experimental results show that the proposed algorithm not only enhances the image brightness and contrast significantly, but also avoids color distortion and over-enhancement in comparison with some other state-of-the-art research papers. So, it effectively improves the quality of sensor images.

## 1. Introduction

Visual information accounts for 83% of the total amount of information that is available to humans. As an extension of visual system, image sensors [1] have key role in our daily life. In general, as image sensors are high integration, low energy consumption and long life, they are widely used in consumer electronics, transportation, medical imaging, astronomical observation and military. However, the captured image quality is strongly dependent on the environment conditions. For example, images captured by sensors under low-light condition, are usually degraded that means having low brightness, low contrast, being blurred or loose the image details. The mentioned drawbacks in image sensors make it difficult for human to distinguish and recognize objects, and brings severe challenges to the subsequent high-level tasks such as target detection [2], video surveillance and so on. Although some cameras have been developed for the low-light environment, there are still some limitation in large scale usage; they are too expensive, they need strict requirements for sensors and lenses. Therefore, using and developing image processing technology for low-light sensor image improvement of the real world is obligatory.

Image enhancement aims to recover real scene information under low illumination in sensor images through relevant technical means and methods, to obtain complete details, structural information and natural, clear images. Low-light image is essentially due to low illumination. In most real-world low illumination images, the uneven light distribution encounters the brighter and darker areas in the image. How to enlarge the pixel value of dark region and simultaneously keep constant the pixel value of brighter region is a challenge. For low-light sensor image improvement, many algorithms have been proposed.

At present, the low-light sensor image enhancement methods are mainly grouped into three categories. The first one is methods based on histogram equalization (HE) [3] where the basic idea is to keep the relative relation between pixel values and make them obey uniform distribution. The original HE methods stretch all the gray levels of the histogram equally to improve the global contrast, later on, some methods performed the local processing. For example, the contrast-limited adaptive histogram equalization (CLAHE) [4] prevents the over-enhancement by restricting the stretch of similar gray levels in local image patches. These methods improve the low-light image and speed up the processing as well, but their drawback is color shift phenomenon. In addition, the image details is lost due to the grayscale merging. The second group are methods based on Retinex model [5], that it is currently the most popular low-light sensor image enhancement algorithm. According to the Retinex model, every captured image by sensors is composed of reflectance and illumination components. By discriminating the two components, the illumination of the original low-light image is removed and the reflection is realized for the image enhancement. Therefore, estimating the illumination component is the core step of any Retinex algorithms. The multi-scale Retinex (MSR) [6] and multi-scale Retinex with color restoration (MSRCR) [7] are two typical algorithms. For this purpose, i.e., discriminating the two components, recently, some new algorithms have been proposed. Fu et al. [8] offered a weighted variational, Guo et al. [9] proposed a simplified enhancement model LIME and achieved good results. LIME always needs a gamma correction for non-linearly rescaling the refined illumination map. This additional post-processing stage defects the model robustness, and causes severe color distortion in some special scenes [10]. The third group are methods based on dehazing model. Dong et al. [11] found that the low-light image is similar to a hazed image after inversion, so they proposed enhancement through the dehazing model. Although, the methods based on dehazing model improve the low-light images visually, they are prone to edge effects and tend to produce unrealistic results. In addition, some algorithms transform the RGB image into other color spaces to achieve image enhancement [12,13], and avoid color distortion whereas, the brightness and contrast still need to be further improved.

Recently, deep neural network has got the dramatic improvements in image classification [14], detection [15], tracking [16] and so on. Deep learning is to establish a network model similar to human brain information processing mechanism. It uses efficient learning strategies to extract features step by step in order to fit the complex nonlinear functions. However, at present, low-light image enhancement methods based on deep learning still need research development and improvement. As far as we know, there are few related documents. Lore et al. [17] tried to train the Stacked Sparse Denoising Auto-Encode (SSDA) to identify signal features from synthetic low-light images and corresponding brighten images. Definitely, after appropriate training, the artificial neural network can enhance the low-light images as well. As an outstanding representative of deep learning, the Convolutional Neural Network (CNN) need few training parameters and thereby simple training procedure. In addition, they are inherently robust to various distortions and invariant images such as zoom, rotation and translation because of local receptive field, weight sharing and pooling.

In this paper, we propose a method based on the color model transformation and the convolution neural network for low-light sensor image enhancement. At first, the low-light image is converted from RGB to HSI color space. Then the hue component (*H*) is kept constant, the saturation component (*S*) is enhanced by using the segmentation exponential method, and the intensity component (*I*) is improved by the Deep Convolution Neural Network (DCNN). Finally, the output is transformed into the RGB space to obtain the enhanced image. Comparing the experimental results with different methods, in terms of subjective and objective criteria, approve that the proposed method outperforms other state-of-the-art methods.

As a conclusion, in following, we refer to the main contributions of this paper. (1) Overcome the shortage of training samples by proposing a simple and time-consuming method for synthesizing the low-light images based on Retinex model. (2) Enhance the low-light sensor images and avoid the color distortion by applying the segmentation exponential algorithm to enhance the saturation (*S*), and using specially designed end-to-end DCNN to improve the intensity (*I*) which can learn the mapping between the intensity of low-light image and the intensity of bright image after training. (3) Improve the low-light synthetic and real images as well.

The paper is organized as follows: In Section 2, we review Retinex model, HSI color space and classical convolutional neural network. In Section 3, the overall framework of our algorithm is explained, where the saturation (*S*) component is improved by segmentation exponential method and the intensity (*I*) component by DCNN. In Section 4, we show the effectiveness of our method in improving brightness, contrast, and preserving the original image color. Finally, in Section 5 we have conclusion and give the future research work.

## 2. Related Works

In this section, we first give Retinex model, then briefly introduce HSI color model, and finally review the basic theory of classical Convolutional Neural Network.

### 2.1. Retinex Model

**Figure 1:**
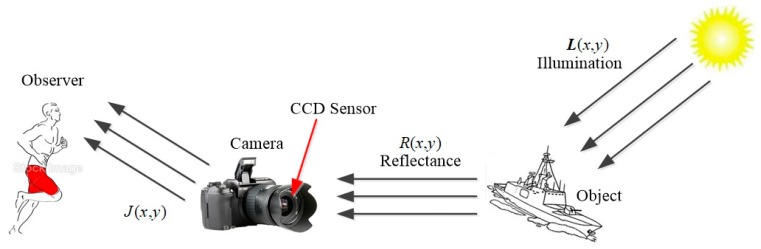
Retinex model.

Retinex theory [5] was first proposed in 1970s and it is now widely used for enhancement of the low-light images. Figure 1 shows the process of an image capturing by a Charge Coupled Device (CCD) sensor, the object is illuminated by sunlight, the reflected rays from the object surface is captured by CCD camera and a human vision system see that object.

In Figure 1, L(x,y) refers to the solar illumination component, R(x,y) is the reflected light rays from the object surface, and J(x,y) indicates the observed image which is captured by a CCD camera. The physical model can be described by the following mathematical functions:

(1) J(x,y)=L(x,y) R(x,y) 

The physical meaning of the above formula can be simply expressed as that the observed image (i.e., low-light image) is equal to the product of the reflectance of image R(x,y) (i.e., desired illuminated image) and the illumination component L(x,y) (i.e., degree of darkness which degrades input image).

### 2.2. HSI Color Model

**Figure 2:**
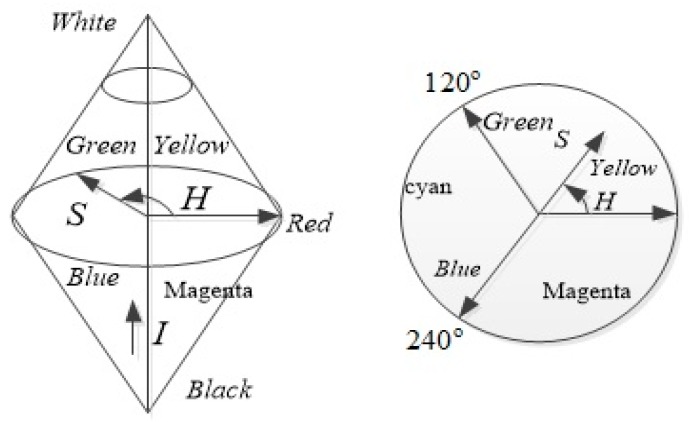
HSI color model.

At present, most of the color image enhancement algorithms process the three color channels independently, i.e., red (*R*), green (*G*) and blue (*B*), though the *R*, *G* and *B* components are strongly correlated. In general, color distortion is occurred if the ratio of the three components change. HSI model uses hue (*H*), saturation (*S*) and intensity (*I*) to represent color images and it is compatible with human visual system as well [18]. The HSI model representation is shown in Figure 2.

Hue represents the feeling of human sense to different colors. Saturation indicates the purity of the color that means the greater saturation is equivalent to the brighter color. Intensity is the brightness of the color. The angle range of the hue is [0°, 360°], in which 0°, 120°and 240° represent red, green and blue colors, respectively. HSI color space has two important characteristics; (1) changing *I* component does not have any effect on the image color information, (2) *H* and *S* components are closely related to the way people feel colors, which is more in line with the perceptual characteristics of human visual perception of the outside world. However, both HSI and RGB color spaces are just different representations of the same physical quantity. The conversion relationship from RGB to HSI are given in following:

(2) $$H=\{\begin{array}{ll} \theta & B\leq G\\ 360^{0}-\theta & B>G \end{array}\;$$

(3) $$S=1-\frac{3\min\left(R,G,B\right)}{R+G+B}\;$$

(4) $$I=\frac{R+G+B}{3}\;$$

where $\theta =\arccos(\frac{(R-G)+\left(R-B\right)}{2{\left[{\left(R-G\right)}^{2}+\left(R-B\right)\left(G-B\right)\right]}^{1/2}})$.

### 2.3. Convolutional Neural Network Model

**Figure 3:**
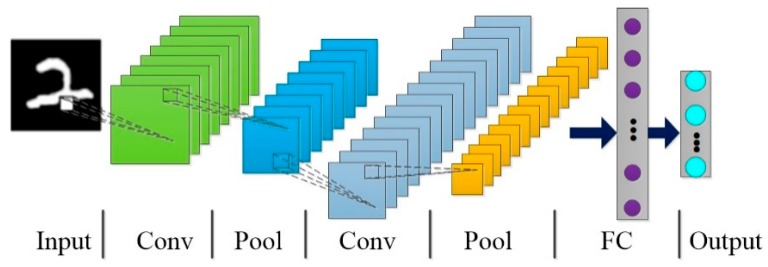
The typical structure of Convolutional Neural Network (CNN).

As shown in Figure 3, the typical convolutional neural network is generally composed of input (Input), convolutional layers (Conv), pooling layers (Pool), fully-connected layer (FC) and output (Output) layer [19].

Several different convolution kernels are usually used in order to extract different features called feature maps of the input data. The convolution process can be described as follows:

(5) $$x_{j}^{l}=f(\sum_{i\in M_{j}}x_{i}^{l-1}*W_{ij}^{l}+b_{j}^{l})\;$$

where $x_{j}^{l}$ and $x_{i}^{l-1}$ represent the feature map of the output of the *j*-th channel of the convolution layer l and the output of the previous layer respectively; $W_{ij}^{l}$ is the convolution kernel connecting the *j*-th feature map of the convolution layers l and l−1, $b_{j}^{l}$ is the bias of the *j*-th feature map of the layer l, $M_{j}$ represents the subset of any current input feature map, ‘∗’ refers to the convolution operation and *f* indicates an activation function.

To reduce the dimension of the feature maps and keep the characteristic scale invariant, the pool layer follows a down-sampling rule for the feature maps. After the alternate transfer of multiple convolution layers and pooling layers, the output is the features for image recognition through the fully-connected layer.

## 3. Proposed Method

In this part, we first give the overall framework of our algorithm, then introduce how to enhance the saturation component *S* and finally give the structure design of the proposed DCNN model and how to enhance the intensity component *I* in detail.

### 3.1. The Overall Framework of Our Mehod

**Figure 4:**
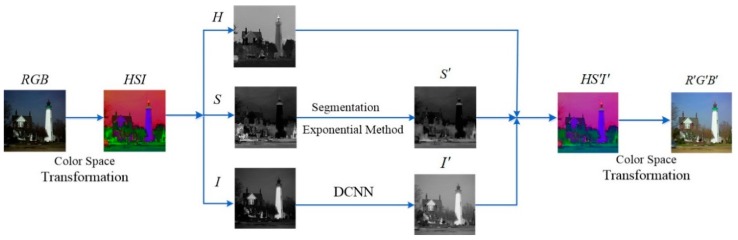
The flow of our method.

In order to overcome the drawback of the current low-light image enhancement algorithms, in this paper, we propose a method based on the color model transformation and the convolution neural network. At first, the low-light image is converted from RGB to HSI color space. Then the hue component (*H*) is kept constant, the saturation component (*S*) is enhanced by using the segmentation exponential method, and the intensity component (*I*) is improved by the convolution neural network. Finally, the output is transformed into the RGB space to obtain the enhanced image as well. The flow chart of our algorithm is shown in the Figure 4.

### 3.2. Saturation Component Enhancement

Generally, the saturation component enhancement algorithms are simple linear transformations. In this paper, we use the segmentation exponential enhancement which is a nonlinear algorithm that can deal with the saturation of different regions separately to achieve better visual effects. According to the saturation component, three regions called as high, medium, and low are defined. If the saturation component image is enhanced globally, the region with high saturation in the original image will be over-saturated, which will cause color distortion. Therefore, when we increase the saturation, we need to consider the low, medium and high areas. For cases, x>0, $e^{x}-1>x$, and $\underset{x\to 0}{lim}\frac{e^{x}-1}{x}=1$, when x is rather small, $e^{x}-1$ is equivalent to x. When the pixel value of the saturation component image is below 0.3, we can think of it as low saturation, which is stretched by exponential transformation; When the pixel value is in the range [0.3,0.7], we can think of it as medium saturation, which needs only proper adjustment; When the pixel value is greater than 0.7, we can think of it as high saturation, which needs to be reduced. The segmentation exponential enhancement algorithm is expressed specifically in following:

(6) $$S^{\prime }(m,n)=\{\begin{array}{ll} a(e^{S(m,n)}-1), & S(m,n)\leq 0.3\\ e^{S(m,n)}-1, & 0.3\leq S(m,n)\leq 0.7\\ b(e^{S(m,n)}-1) & else \end{array}\;$$

where S and $S^{\prime }$ are the saturation before and after enhancement; a and b are used to adjust the transformation scale. In this experiment process we found that the best enhancement effect can be achieved when a takes 1.1~1.3 and b takes 0.5~0.7. In this paper, we decide to use the parameters in the literature [12], that is, a=1.3, b=0.7.

### 3.3. Intensity Component Enhancement

Literature [20] suggests that learning the mapping relationship between rainy image and rain (i.e., residual of the rainy image and clean image) can reduce the pixel range of the mapping and make the learning process easier. Inspired by this knowledge, we do not directly use the deep convolution neural network to train and learn the end-to-end mapping relationship between low-light RGB image and normal illumination RGB image, but we do learn the mapping of the intensity component between low-light image and normal illumination image in HSI space. As for RGB, it still needs to consider the color changes during training, we use HSI color space and achieve more conducive of network training. So, this model inputs the intensity component of the low-light image and outputs the enhanced intensity component.

#### Network Architecture

**Figure 5:**
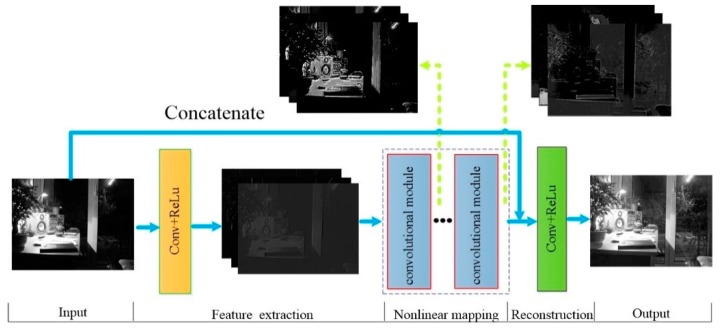
The network structure of DCNN for enhancing intensity.

Unlike traditional convolution neural network, our model called DCNN used to enhance intensity has no pooling layer and fully-connected layer. According to different functions, five parts can be considered; input, feature extraction, nonlinear mapping, reconstruction, and output. As seen in Figure 5, feature extraction is mainly used for the edge texture and other structural features of image, whereas the non-linear mapping basically targets the high-light region first and then the low-light region.

The network model requires that the network output and input image have the same size. But both the pooling layer and the convolution operation make the size become smaller. That means, the original image greatly loses information. Therefore, zero-padding operation is performed before all the convolution layers, which guarantee the image size during the forward transmission of the network is not changing at all. In following, the five parts mentioned in the network are explained in detail.

(1) Input and output: To facilitate better training of the network, neither the input nor output of the proposed network is a whole image. In contrast, patches of low-light image and corresponding normal illumination image are randomly selected as training samples. At the same time, the used model only enhances the intensity component, so the input is the intensity component of the low-light image and the output is the enhanced intensity component.

(2) Feature extraction: The first part of this network is designed for feature extraction and made up of convolution layer. The convolution layer is often used to extract the features of different local regions by convolution operation between several learnable filters and input image or upper layer feature maps and the nonlinear activation function. Here, we use $H_{i}$ to represent the first i layer feature map of the convolutional neural network; suppose the input image patch of the network is X and the feature extraction function is expressed as,

(7) $$H_{1}(X)=max(0,W_{1}*X+b_{1})\;$$

where $W_{1}$ denotes the filters, $b_{1}\in R^{n_{1}}$ represents the bias vector, and X is a matrix with size n×n. Suppose $f_{1}$ with size of a single filter and c as the number of channels in the input image (we explained that only the intensity component is processed, so *c* = 1), if there are $n_{1}$ filters, then the size of $W_{1}$ is $f_{1}\times f_{1}\times n_{1}\times c$.

**Figure 6:**
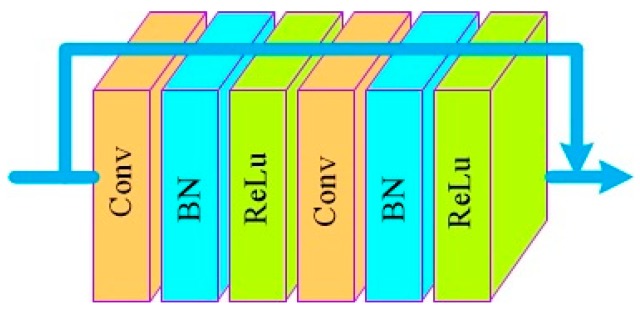
The model of convolutional module.

(3) Nonlinear mapping: In this paper, nonlinear mapping consists of several convolutional module including two convolution layers, two batch normalization (BN) layers and two ReLu [21] layers. The convolutional module is shown in the Figure 6. Inspired by DenseNet [22], our model uses feature maps connection mechanism, that is, each convolutional module combines its own input feature maps and the output feature maps together as the input to the next layer, so that feature maps of each layer can be utilized by all the later layers.

The BN algorithm normalizes the output of all the intermediate layers in CNN. So, it deducts the impact on changes of the network data distribution in the training phase of the neural network parameters. Thereby, using BN algorithm speeds up the network convergence and stability [23].

Supposed that $\left\{\mu_{1},\cdot \cdot \cdot ,\mu_{n}\right\}$ is the input data of any intermediate layer of the model, we first obtain their mean and variance respectively as follows.

(8) $$E\left[\mu \right]=\frac{1}{m}\sum_{i=1}^{m}\mu_{i}\;$$

(9) $$var\left[\mu \right]=\frac{1}{m}\sum_{i=1}^{m}{\left(\mu_{i}-E\left[\mu \right]\right)}^{2}\;$$

Then, the data is normalized to data with a mean of 0 and a variance of 1.

(10) $${\hat{\mu }}_{i}=\frac{\mu_{i}-E\left[\mu \right]}{\sqrt{var\left[\mu \right]+\varepsilon }}\;$$

where ε is a constant close to 0.

Last, the reconstruction parameters α and β are introduced to reconstruct the data after batch normalization and get the final output.

**Figure 7:**
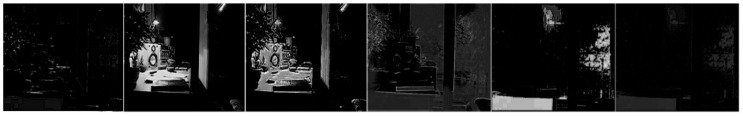
The feature maps learned in the nonlinear mapping part.

The nonlinear mapping part can map the features of the former part from the low-dimensional space to the high-dimensional space. This process is written in following

(11) $$H_{k-1}(X)=\max(0,\mathrm{BN}(W_{k-1}*H_{k-2}(X)+b_{k}))\;k\geq 3\;$$

where k indicates the depth of the proposed network, equivalently, the k−1 layer is the last layer of the nonlinear mapping part. In order to investigate what the model has learned, we visualize the feature maps in the middle process after training. We randomly select six learned feature maps as shown in Figure 7. Observations prove that the first several layers focus on the high-light regions and the latter layers focus on low-light regions.

(4) Reconstruction: In this paper, the low-light image is enhanced based on the reconstruction concept. Reconstruction is to minimize the corresponding intensity component error between the model output and the normal illumination image (namely, ground truth) by training. The original input is considered to contain more details [24]. Therefore, we use concatenation to combine the feature maps of the last ConvBlock and the input image, so that the original information can be preserved for detail restoration. In this part, only one convolution layer is needed, see Equation (11).

(12) $$H_{k}(X)=W_{k}*(H_{k-1}(X),X)+b_{k}\;$$

In order to automatically learn the network model parameter $\theta =\left\{W_{1}\cdots W_{k};b_{1}\cdots b_{k}\right\}$, the loss function in Equation (12) can be minimized by back propagation algorithm and stochastic gradient descent method. Assuming a training sample set as $D=\left\{\left(X_{1},Y_{1}),\cdots (X_{i},Y_{i}\right)\right\}$.

(13) $$L=\frac{1}{N}\sum_{i=1}^{N}{\left\|H_{k}(X_{i})-Y_{i}\right\|}_{F}^{2}+\lambda \sum_{k=1}^{k}{\left\|W_{k}\right\|}_{F}^{2}\;$$

where $X_{i}$ and $Y_{i}$ are the samples of the low-light image intensity component in the *i*-th input and that of the corresponding ground truth, *N* is the number of training samples, and λ as a weight constraint term prevents the over-fitting.

## 4. Experiments Results

### 4.1. Dataset Generation

At present, low-light image enhancement methods based on deep learning still need development and improvement. As, deep learning requires a large number of training samples, it is difficult to take low-light image and ground truth in the same scene. Therefore, we propose a generation method of training samples, which is simple for implementation and also less time-consuming.

According to Equation (1), the low-light image is the product of the illumination and the reflection component (namely, ground truth). In order to cover as many low-light images as possible, we have collected 500 images which captured by high quality sensors from the BSD dataset [25] as reflective component, then a total of 256,000 image patches with size 40 × 40 are randomly selected for training. Assume the illumination component *L* with the uniform distribute, L∼U(0,1), then the synthetic low-light image patch can be represented as J(x,y)=L⋅R(x,y).

### 4.2. Parameter Settings

We use the MatConvNet [26] software package to train the proposed model. All the experiments are carried out by using MatLab softwate (R2014a), on a PC with 16G RAM, an Intel (R) Core (TM) i7-7700 CPU 3.30GHz and an Nvidia GeForce GTX1070 GPU.

In this paper, the proposed network depth is k=7, the size of a single convolution kernel is 3×3 and the feature extraction part has 64 filters of size 3×3×64. The nonlinear mapping part has 64 filters of size 3×3×64×64. The reconstruction part only needs one filter with size 3×3×64. The filters of all network layers adopt the initialization method proposed by He et al. [27] and the biases are set to 0. Adam algorithm with a batch size of 128 is used to train 50 Epochs for our proposed model. The initial learning rate is 0.1 and it will be reduced by 10 times every 10 Epochs.

### 4.3. Study of DCNN Parameters

**Figure 8:**
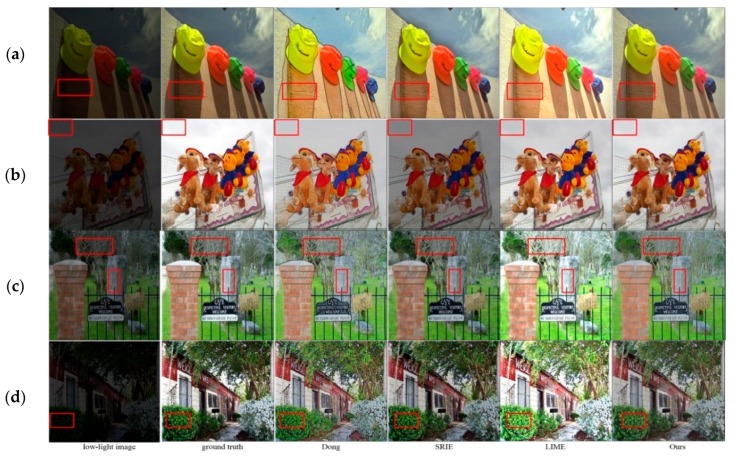
The subjective visual comparison of different algorithms for synthetic low-light images. (**a**) “caps” from LIVE1, (**b**) “carnivaldolls” from LIVE1, (**c**) “cemetry” from LIVE1, (**d**) “building2” from LIVE1.

**Figure 9:**
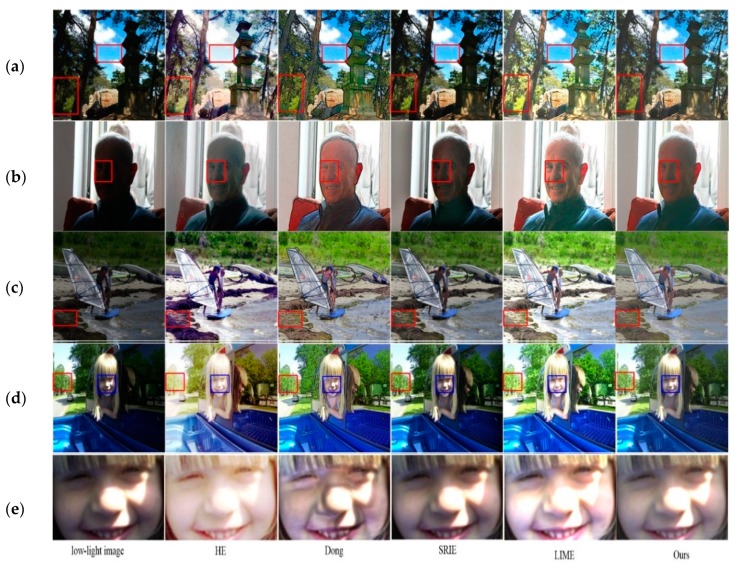
The subjective visual comparison of different algorithms for real low-light images. (**a**) the imae from DICM, (**b**) The image from VV dataset, (**c**) The image from NASA dataset, (**d**) The image from NASA dataset, (**e**) the result of partial enlargement of (**c**).

As mentioned before, the proposed network structure does not have any pooling layer, so the model parameters mainly include the number of network layers and the size and the quantity of filters. We use the filters of size 3×3, which is used in most cases. We have also experimented the number of filters and the network layers. Table 1 represents the obtained PSNR of the output to enhance Figure 8a and LOE of the output to enhance Figure 9e after 50 Epochs of training. 

Referring to the method of setting up the number of filters in the SRCNN [28], we have set $n_{1}=64,n_{i-1}=32$ and $n_{1}=64,n_{i-1}=64$ in this paper for comparative analysis. Where, $n_{1}$ represents the number of filters in the first convolution layer and $n_{i-1}(i\geq 3)$ is the number of filters in all the later convolution layers. The network layers are 5, 7 and 9 in order.

As shown in Table 1, increasing the number of filters outperform the performance as well, though it is engaged with the computational complexity. We also found that the maximum PSNR of the output image is achieved for the network structure with 7 layers. Also increasing the number of network layers does not necessarily improve the performance, because (1) defects of gradient dispersion, (2) repeating a simple original network may results in unreasonable network architecture.

### 4.4. Comparison with State-of-the-Art Methods

In order to evaluate the proposed method, we use both synthetic and real low-light images. Then the results are compared with HE [29] algorithm and other recent state-of-the-art low-light image enhancement methods such as Dong, SRIE and LIME in terms of subjective and objective criteria.

#### 4.4.1. Evaluation on Synthetic Test Dataset

In this paper, a total of 29 synthetic low-light images as the test samples are selected from the open dataset LIVE1 [30]. Figure 8 shows the results for four synthesized low-light images, which marked as (a)–(d) from top to bottom. Where, (a), (b) are uniformly illuminated low-light images, and (c), (d) are unevenly illuminated images. As, it is shown in Figure 8 for (a), (c) and (d), Dong method always generates serious black edge. For example, in Figure 8a, originally under the hat, a shadow is seen that indicates, serious contour lines appear after enhancement. Although, SRIE method preserves the image color and no false contour is appeared, but there is a limitation for the brightness improvement. LIME method produces over-enhanced results for bright regions and it possibly leads to different degrees of color distortion. For example, in Figure 8c,d, the flowers and plants are yellowish, whereas in ground truth they are green. The proposed algorithm not only improves the image brightness and contrast, but also preserves the image color information too close to the ground truth image. Meanwhile, like other algorithms, it is not able to enhance the dark regions of a large white area in the actual scene. 

As ground truth is known for the used synthetic low-light images, we can compare the performance of different algorithms in terms of objective criteria. So, for performance evaluation, peak signal-to-noise ratio (PSNR), structural similarity (SSIM), mean square error (MSE) and lightness order error (LOE) [31] were used. PSNR reflects the distortion degree of an image, so high PSNR value indicates less distortion. SSIM characterizes the integrity of image structure information, so high SSIM value shows more similar structural features between the enhanced image and the corresponding ground truth. MSE reflects the difference between the enhanced image and the ground truth, so the small MSE means that the enhanced image is similar to the original normally illuminated image. LOE mainly evaluates the naturalness preservation ability of the enhanced image, so the small LOE value indicates that the brightness order of the image is reasonably preserved and the image is more natural. Table 2 gives the average objective parameters for different enhancement algorithms where LIVE1 is used as the test dataset.

According to the results written in Table 2, the proposed algorithm outperforms other approaches in terms of PSNR, MSE and LOE, only it got less SSIM sin comparison with SRIE algorithm. The achievements indicate that the enhanced image by our algorithm is much closer to the original image, it is more realistic and natural, and the distortion is small.

#### 4.4.2. Evaluation on Real-Word Dataset

In order to show the proposed algorithm capability for the enhancement of not only the synthetic low-light images but also for actual low-light images, 17 typical images were selected from NASA [32], DICM [33] and VV [34] dataset. All chosen images are real low-light images taken by commercial sensor cameras. The experimental results are shown in Figure 9 and written in Table 3.

For subjective evaluation, in Figure 9, the four enhanced images listed as (a)–(e) from top to bottom are shown. Images a,c,d show that, the HE algorithm can improve the overall brightness and contrast, but serious color deviation occurs due to the super saturation. The image color is not changed by Dong algorithm, but serious edge effect has occurred. Because, Dong algorithm uses defogging method for image enhancement, where the low-light image after inversion is not exactly the same as a foggy image. Therefore, Dong algorithm is not to able to estimate the atmospheric light value of pseudo-foggy images. Moreover, SRIE algorithm is appropriate for slightly dark regions, and it is not recommended for the brightness improvement of dark regions where the details cannot be easily distinguished. Although LIME algorithm improves the brightness of dark regions, its main drawbacks are over-enhancement and color distortion [35,36]. As shown in Figure 9a, the leaves and the stone tablet have the color distortion. In addition, the right half face of the girl in Figure 9e is originally exposed. LIME algorithm takes the same degree of enhancement whether the region in the original low-light image is normal or dark. So we can see that the right half of the face has been excessively enhanced, which is not consistent with human’s visual perception. Our proposed algorithm can well maintain the color and avoid the over-enhancement, but it is really not as good as the LIME algorithm in enhancing brightness, especially for a particularly dark area.

Obviously, there is no ground truth or reference for the real low-light images, that means the objective quality evaluation with no reference. In this paper, information entropy, color change degree, and LOE are used to evaluate the enhanced images in terms of objective criteria. The information entropy indicates the scale of the image information, the large value shows rich image with more details. The degree of changing hue in the enhanced image reflects the color preservation, i.e., the small value indicates the low color distortion. VIF is an image quality evaluation index which was proposed based on the natural image statistical model, image distortion model, and human visual system model. The greater the value of VIF, the better the image quality is [37,38]. Table 3 gives the average values of various objective evaluation indexes for enhancing 17 low-light images by different algorithms.

According to the results written in Table 3, HE and Dong have higher color change values, they have the worst capacity for color retention. LIME has higher information entropy which shows that the algorithm preserves the image information as well, but the corresponding LOE value is large, that indicates the order of image brightness has been destroyed and the naturalness is poorly retained. Moreover, SRIE algorithm has the lowest information entropy. However, according to the three objective parameters, except the information entropy in comparison with LIME algorithm, the proposed approach outperforms other methods for image enhancement.

**Figure 10:**
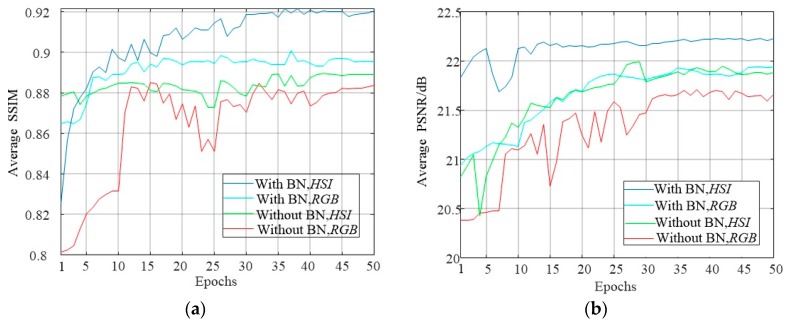
The convergence performance for SSIM and PSNR of HSI and RGB methods under BN and without BN. (**a**) The average SSIM within 50 Epochs, (**b**) The average PSNR within 50 Epochs.

Now, for further verification and comparing the performance of the proposed algorithm and the algorithm for direct RGB image enhancement, we implement two groups of control experiments on the test dataset LIVE1 after training 50 Epochs under the condition of BN and without BN. And then we record the average PSNR and SSIM obtained during the network testing in each Epoch as shown in the Figure 10.

As seen in Figure 10, the HSI training method converges rapidly in comparison with direct enhancement of the RGB image and it gets the greater values in average for PSNR and SSIM with BN or without BN. At the same time, it can also be seen that the batch normalization can also improve the convergence speed of the network training as well.

### 4.5. Application

**Figure 11:**
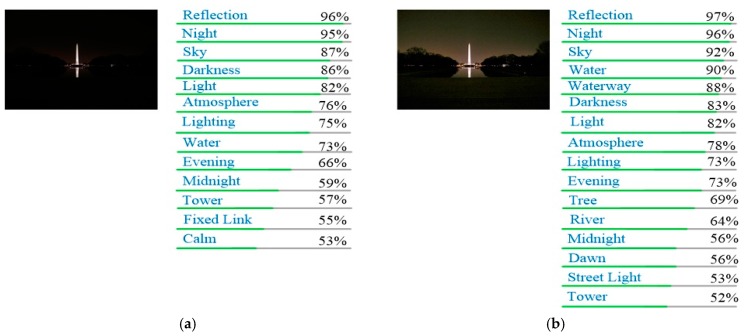
Results of Google Cloud Vision API for “27” from DICM dataset. (**a**) Recognizing result of low-light image; (**b**) Recognizing result of our enhanced image.

**Figure 12:**
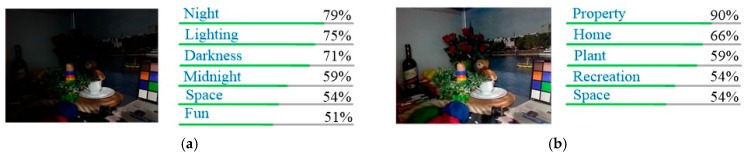
Results of Google Cloud Vision API for “Room” from LIME dataset. (**a**) Recognizing result of low-light image; (**b**) Recognizing result of our enhanced image.

To provide further evidence that our method could be useful for computer vision applications, we use Google Vision API (https://cloud.google.com/vision/) to test whether using our outputs can improve the recognition performance. The results are shown in Figure 11 and Figure 12.

As can be seen, using our enhanced image, the recognition of API is better than original low-light image. For example, in Figure 11, the API does not recognize trees in the original image, but trees are identified with our enhancement method. Another example is shown in Figure 12, where the original image is marked as “lighting” and “darkness” by the API, and after enhancement, the plant in the image is marked.

## 5. Conclusions

The bottleneck of mainstream low-light image enhancement algorithms is the color distortion. In order to overcome the mentioned problem, in this paper, at first, we consider human visual characteristics, transform the low-light image from RGB to HSI color space where the hue component *H* is kept constant. Then we use the segmentation exponential method to stretch the saturation component *S*, make full use of the powerful learning ability of the deep convolution neural network extracting the intrinsic features from massive data and fitting complex functions to learn end-to-end mapping relationship between the intensity component of low-light and ideal-light image thus obtain an enhanced intensity component. Finally, we transform the enhanced HSI image to the RGB color space. The experimental results according to the synthetic and real low-light images in terms of both objective and subjective criteria show that the proposed algorithm not only improves the brightness and contrast, but also avoids the color distortion as well. In the future, we will continue to optimize the network model to further improve the brightness of the dark area. 

## Figures and Tables

**Table 1:** PSNR of different network layers and filters numbers.

Layers	Filters Numbers	PSNR	LOE
5	$n_{1}=64,n_{i-1}=32$	21.74	401
5	$n_{1}=64,n_{i-1}=64$	21.87	394
7	$n_{1}=64,n_{i-1}=32$	22.23	371
7	$n_{1}=64,n_{i-1}=64$	22.31	365
9	$n_{1}=64,n_{i-1}=32$	22.04	383
9	$n_{1}=64,n_{i-1}=64$	22.17	378

**Table 2:** Objective evaluation of different algorithms for synthetic low-light images.

Method	HE	Dong	SRIE	LIME	Ours
PSNR	16.19	16.29	21.08	13.47	21.33
SSIM	0.7985	0.7947	0.9579	0.8097	0.9204
MSE	1928.3	1699.6	686.4	3230.7	389
LOE	505	2040	776	1277	402

**Table 3:** Objective evaluation index of different algorithms for real low-light images.

Method	HE	Dong	SRIE	LIME	Ours
entropy	7.1412	6.9541	6.9542	7.2612	7.1425
Chromaticity change	0.2474	0.0313	0.0045	0.0124	0.0032
LOE	571	1484	972	1390	378
VIF	0.4782	0.4262	0.6153	0.3444	0.7356

## References

[1] Helmers H., Schellenberg M. (2003). CMOS vs. CCD sensors in speckle interferometry. Opt. Laser Technol..

[2] Tan Y., Li Q., Li Y., Tian J. (2015). Aircraft detection in high-resolution SAR images based on a gradient textural saliency map. Sensors.

[3] Pizer S.M., Amburn E.P., Austin J.D., Cromartie R., Geselowitz A., Greer T., ter Haar Romeny B., Zimmerman J.B., Zuiderveld K. (1987). Adaptive histogram equalization and its variations. Comput. Vis. Graph. Image Process..

[4] Pisano E.D., Zong S., Hemminger B.M. (1998). Contrast Limited Adaptive Histogram Equalization image processing to improve the detection of simulated spiculations in dense mammograms. J. Digit. Imaging.

[5] Edwin H.L. (1977). The retinex theory of color vision. Sci. Am..

[6] Zhang S., Wang T., Dong J.Y., Yu H. (2017). Underwater Image Enhancement via Extended Multi-Scale Retinex. Neurocomputing.

[7] Jobson D.J., Rahman Z., Woodell G.A. (1997). A multiscale retinex for bridging the gap between color images and the human observation of scenes. IEEE Trans. Image Process..

[8] Fu X.Y., Zeng D., Huang Y., Zhang X.P., Ding X.H. A weighted variational model for simultaneous reflectance and illumination estimation. Proceedings of the 2016 IEEE Conference on Computer Vision and Pattern Recognition (CVPR).

[9] Guo X.J., Li Y., Ling H.B. (2017). LIME: Low-Light Image Enhancement via Illumination Map Estimation. IEEE Trans. Image Process..

[10] Hao S., Feng Z., Guo Y. (2018). Low-light image enhancement with a refined illumination map. Multimed. Tools Appl..

[11] Dong X., Wang G., Pang Y., Li W.X., Wen J.T., Meng W., Liu Y. Fast efficient algorithm for enhancement of low lighting video. Proceedings of the International Conference on Multimedia & Expo (ICME 2011).

[12] Wu F., Kin T.U. Low-Light image enhancement algorithm based on HSI color space. Proceedings of the 10th International Congress on Image and Signal Processing, Biomedical Engineering and Informatics (CISP-BMEI 2017).

[13] Nandal A., Bhaskar V., Dhaka A. (2018). Contrast-based image enhancement algorithm using grey-scale and colour space. IET Signal Process..

[14] Krizhevsky A., Sutskever I., Hinton G.E. ImageNet classification with deep convolutional neural networks. Proceedings of the 25th International Conference on Neural Information Processing Systems (NIPS).

[15] Ren S.Q., He K.M., Girshick R., Sun J. (2015). Faster r-cnn: Towards real-time object detection with region proposal networks. Proceedings of the 28th International Conference on Neural Information Processing Systems (NIPS).

[16] Wang L.J., Ouyang W.L., Wang X.G., Lu H.C. (2015). Visual tracking with fully convolutional networks. Proceedings of the IEEE International Conference on Computer Vision (ICCV), CentroParque Convention Center.

[17] Lore K.G., Akintayo A., Sarkar S. (2017). LLNet: A deep autoencoder approach to natural low-light image enhancement. Pattern Recognit..

[18] Zhang W.F., Liang J., Ren L.Y. (2017). Fast polarimetric dehazing method for visibility enhancement in HSI colour space. J. Opt..

[19] He S.F., Lau R.W., Liu W.X. (2015). SuperCNN: A Superpixelwise Convolutional Neural Network for Salient Object Detection. Int. J. Comput. Vis..

[20] Fu X., Huang J., Zeng D. Removing Rain from Single Images via a Deep Detail Network. Proceedings of the 2017 IEEE Conference on Computer Vision and Pattern Recognition (CVPR).

[21] Glorot X., Bordes A., Bengio Y. Deep sparse rectifier neural networks. Proceedings of the 14th International Conference on Artificial Intelligence and Statistics (AISTATS).

[22] Huang G., Liu Z. Densely Connected Convolutional Networks. Proceedings of the 2017 IEEE Conference on Computer Vision and Pattern Recognition (CVPR).

[23] Ioffe S., Szegedy C. (2015). Batch Normalization: Accelerating Deep Network Training by Reducing Internal Covariate Shift. PMLR.

[24] Li J., Feng J. (2017). Deep convolutional neural network for latent fingerprint enhancement. Signal Process. Image Commun..

[25] Xie S., Tu Z. (2015). Holistically-Nested Edge Detection. Int. J. Comput. Vis..

[26] Vedaldi A., Lenc K. Matconvnet: Convolutional neural networks for matlab. Proceedings of the 23rd Annual ACM Conference on Multimedia Conference.

[27] He K., Zhang X., Ren S. Delving Deep into Rectifiers: Surpassing Human-Level Performance on ImageNet Classification. Proceedings of the 2015 IEEE International Conference on Computer Vision (ICCV) ICCV ’15.

[28] Dong C., Chen C.L., He K.M. (2016). Image Super-Resolution Using Deep Convolutional Networks. IEEE Trans. Pattern Anal. Mach. Intell..

[29] Gonzalez R.C., Woods R.E. (2017). Digital Image Processing.

[30] Sheikh H.R., Sabir M.F., Bovik A.C. (2006). A Statistical Evaluation of Recent Full Reference Image Quality Assessment Algorithms. IEEE Trans. Image Process..

[31] Wang S.H., Zheng J., Hu H.M. (2013). Naturalness Preserved Enhancement Algorithm for Non-Uniform Illumination Images. IEEE Trans. Image Process..

[32] NASA Retinex Image Processing. http://dragon.larc.nasa.gov/retinex/pao/news/.

[33] Lee C., Kim C.S. Contrast enhancement based on layered difference representation. Proceedings of the 19th IEEE International Conference on Image Processing.

[34] Vassilios Vonikakis. https://sites.google.com/site/vonikakis/datasets.

[35] Wang S., Luo G. (2018). Naturalness Preserved Image Enhancement Using a priori Multi-Layer Lightness Statistics. IEEE Trans. Image Process..

[36] Liu J.Y., Yang W.H. Joint Denoising and Enhancement for Low-Light Images via Retinex Model. Proceedings of the 14th International Forum on Digital TV and Wireless Multimedia Communication.

[37] Chow L.S., Rajagopal H., Paramesran R. (2016). Correlation between subjective and objective assessment of magnetic resonance (MR) images. Magn. Reson. Imaging.

[38] Ying Z.Q., Li G., Gao W. A Bio-Inspired Multi-Exposure Fusion Framework for Low-Light Image Enhancement. https://github.com/baidut/BIMEF.

